# Interaction of the Human Contact System with Pathogens—An Update

**DOI:** 10.3389/fimmu.2018.00312

**Published:** 2018-02-26

**Authors:** Sonja Oehmcke-Hecht, Juliane Köhler

**Affiliations:** ^1^Institute of Medical Microbiology, Virology and Hygiene, Rostock University Medical Center, Rostock, Germany

**Keywords:** contact system, bradykinin, inflammation, infection, pathogen

## Abstract

The name human contact system is related to its mode of action, as “contact” with artificial negatively charged surfaces triggers its activation. Today, it is generally believed that the contact system is an inflammatory response mechanism not only against artificial material but also against misfolded proteins and foreign organisms. Upon activation, the contact system is involved in at least two distinct (patho)physiologic processes:*i*. the trigger of the intrinsic coagulation *via* factor XI and *ii*. the cleavage of high molecular weight kininogen with release of bradykinin and antimicrobial peptides (AMPs). Bradykinin is involved in the regulation of inflammatory processes, vascular permeability, and blood pressure. Due to the release of AMPs, the contact system is regarded as a branch of the innate immune defense against microorganisms. There is an increasing list of pathogens that interact with contact factors, in addition to bacteria also fungi and viruses bind and activate the system. In spite of that, pathogens have developed their own mechanisms to activate the contact system, resulting in manipulation of this host immune response. In this up-to-date review, we summarize present research on the interaction of pathogens with the human contact system, focusing particularly on bacterial and viral mechanisms that trigger inflammation *via* contact system activation.

## Intrinsic Coagulation Pathway—The Procoagulant Arm of the Contact System

**Figure 1:**
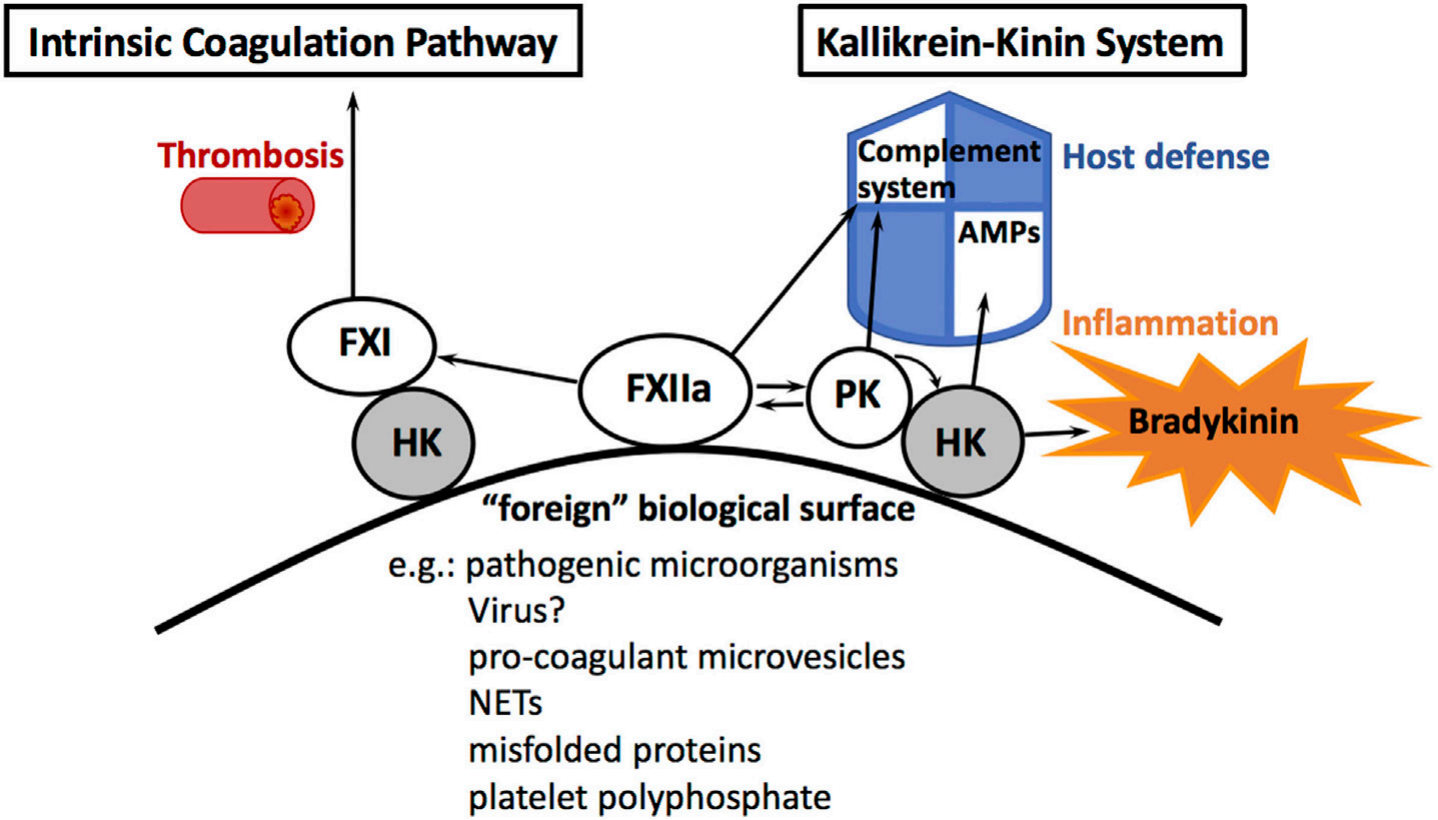
The human contact system. Assembly of contact system factors on foreign biological or artificial surfaces activates factor XII (FXII). FXIIa activates factor XI (FXI) that triggers the intrinsic pathway of coagulation, which is involved in thrombosis. FXII also activates plasma kallikrein (PK), which cleaves high molecular weight kininogen (HK), followed by the release of the pro-inflammatory peptide bradykinin and antimicrobial peptides (AMPs). FXII and PK contribute *in vitro* to complement activation.

The human contact system consists of two proteases, factor XII (FXII) and plasma prekallikrein (PPK) as well as the non-enzymatic cofactor high molecular weight kininogen (HK, see Figure 1). The proteins are produced in the liver and circulate as zymogens in the blood stream or are assembled on endothelial cells, neutrophils, and platelets. When blood is exposed to foreign biological or artificial surfaces, zymogen FXII binds through and autoactivates into an enzyme. Activation is accompanied by a major conformational change in the structure of FXII (1). Classically, it is stated that FXII has to interact with negatively charged surfaces for activation, but the current paradigm is that any artificial surface has the potential for FXII autoactivation (2). HK, which is in a noncovalent complex with PPK (3), also binds to the surface, thereby exposing PPK for activation by FXII cleavage. In turn, activated plasma kallikrein (PK) cleaves and activates more FXII, forming a powerful activation feedback loop. When sufficient amounts of FXII are activated on the surface, FXII activates coagulation factor XI (FXI), leading to subsequent thrombin formation. This result—*in vitro*—in the formation of a fibrin clot and is used as a diagnostic coagulation test—the activated partial thromboplastin time (aPTT). However, individuals with congenital deficiencies in FXII, PPK, or HK, who show a prolonged aPTT, do not have bleeding diathesis or abnormal hemostasis, indicating that the intrinsic coagulation pathway does not contribute to physiological hemostasis (4). Moreover, contact activation *in vivo* always occurs under pathological conditions, such as thrombosis (5), sepsis, or ARDS (6, 7), which makes FXII a promising therapeutic target to limit thrombosis without increasing bleeding risk (8). Thus, it has been questioned whether activation of the intrinsic coagulation by FXII is really its main physiological function. Instead, it was suggested that the pro-inflammatory arm of the contact system—the kallikrein–kinin system—is more related to physiological *in vivo* functions (9).

## The Pro-Inflammatory Kallikrein–Kinin System as a Link to Innate and Adaptive Immunity

High molecular weight kininogen is encoded by the *KNG1* gene, which is alternatively spliced into two products, high and low molecular weight kininogen. High molecular weight kininogen (HK) contains six domains (D1–D6) with a range of procoagulant, pro-inflammatory, or antimicrobial functions. Low molecular weight kininogen (LK) lacks D6, wherefore it cannot bind PK or FXI and does not belong to the contact system. Upon activation by FXII, PK cleaves HK and the nonapeptide bradykinin will be released from D4 (10).

Bradykinin is one of the most potent inflammatory mediators in humans, after binding through its cell receptor B2R (11) it activates signaling pathways resulting in increased vascular permeability, vasodilation, hypotension, pain, fever. Furthermore, kinin receptors appear to be involved in autoimmune diseases (12). Although bradykinin is a short-lived mediator, it stimulates the production of superoxide radicals and nitric oxide and modulates the mobilization and release of histamine, arachidonic acid, prostaglandin E2, pro-inflammatory interleukin-1, and TNF-alpha (13).

Additionally, bradykinin is involved in activation of cellular innate immune responses, such as migration of neutrophils (14) and stimulation of alveolar macrophages (15). Exogenous bradykinin activates immature dendritic cells *via* B2R, thereby stimulating adaptive immunity (16). Moreover, cooperative activation of B2R and toll-like receptor 2 is responsible for an interferon-γ response in dendritic cells, linking innate and adaptive immune responses (17).

It has been proposed that neutrophils interact with the contact system to boost neutrophil extravasation by bradykinin-mediated vasodilatation (18). Moreover, PK and FXII itself trigger inflammation by causing aggregation and degranulation of human neutrophils (19, 20). FXII contributes further to inflammation by induction of pro-inflammatory cytokines from macrophages (21).

Neutrophil extracellular traps (NETs) have been shown to bind and activate contact factors (22). Released from neutrophils in response to infectious and pro-inflammatory stimuli, NETs immobilize invading pathogens within a fibrous matrix consisting of DNA, histones, and antimicrobial peptides (AMPs) (23), providing a suitable surface for contact system activation. The overall consequences of NETs release are not clear, far from being univocal. NETs may also be protective for the invading pathogen and contribute to autoimmune diseases (24).

Recent studies further reveal that also procoagulant microvesicles are equipped with a surface that allows binding and activation of contact factors and bradykinin release (25). Microvesicles are continuously shed from the membrane of every cell type examined to date. Procoagulant microvesicles are shed due to an infectious stimulus from the plasma membrane of monocytes (25, 26). The outer surface of such microvesicles is enriched in phosphatidylserine, which provides a catalytic surface for the assembly of contact and coagulation factors (27). Moreover, microvesicles can explore antimicrobial activity (28), entrap bacteria, and prevent their dissemination from the local focus of infection in an animal model of sepsis (29). Formation and release of procoagulant microvesicles follows the principles of pattern recognition, as activation of monocytes is triggered by the binding of streptococcal M1 protein to toll-like receptor 2 (30), which suggests that microvesicle release is part of the innate immune reaction.

A further link between the contact system and host defense is activation of the alternative complement pathway by FXII that triggers activation of the C1 complex (31). In the alternative complement pathway, PK can replace factor D for the activation of C3 convertase (32, 33). Simultaneous activation of the contact and complement system results in the generation of nascent molecules that have significant impact in various in inflammatory diseases including angioedema and cancer (34). Whether contact system factors trigger activation of the complement system *in vivo* remains to be investigated.

Due to the generation of bradykinin by PK, the cleavage product HKa is formed, which differs from HK because of conformational changes (35). HKa stimulates secretion of the cytokines TNFα, interleukin IL-1β, IL-6, and the chemokines IL-8 and MCP-1 from human mononuclear cells, all of which are known to contribute to the inflammatory process (36).

Finally, HK-derived peptides display potent antibacterial and antifungal properties (37–40), contributing pivotal components of innate immunity, as such AMPs represent a first-line defense against invading pathogens. Recently Cagliani et al. (41) published a phylogenetic analysis indicating that mammalian kininogen genes evolved adaptively, in contrast to the other contact system genes. It has been proposed that kininogen gene *KNG1* has been a target of long-lasting and strong selective pressures, suggesting that kininogen plays a central role in the modulation of immune responses (41).

Taken together, the kallikrein–kinin system contributes to innate immune defense by bradykinin dependent and independent mechanisms. Activation of contact factors triggers inflammatory reactions that potentiate the host response against invading pathogens.

## Binding of Contact Factors at the Pathogen Surface

In order to respond to a broad range of microbes the innate immune system uses a variety of proteins, which recognize surface features of microbial pathogens that differ from human cell membranes. Although the contact system is activated *in vitro* by high doses of purified bacterial lipopolysaccharides (LPS) (42, 43), contact factors bound to specific proteins and virulence determinants on the bacterial surface. Over 20 bacterial species are known to bind and activate contact factors on their surface, but the bacterial binding protein and the activating mechanism is often unknown (44). However, there are certain similarities in structure and property of bacterial proteins, which interact with contact factors. Many bacterial species possess long filamentous structures known as curli, fimbriae, or pili extending from their surfaces (45). Gram-negative bacteria, such as *Escherichia coli* and *Salmonella enterica* subsp. *enterica* ser. Typhimurium express curli fibers that bind all contact factors (46, 47). Curli play a major role in biofilm formation (48) and as adhesins, as they bind to proteins of the extracellular matrix. Similarly, *Porphyromonas gingivalis*, a Gram-negative periodontal pathogen, expresses long peritrichous, filamentous components, known as fimbriae, on the bacterial surface that are implicated in binding of contact factors (49). Interestingly both, curli from *E. coli* or *Salmonella enterica* as well as fimbriae from *P. gingivalis* (50) belong to a class of stable, ordered proteins, characterized structurally by repeating beta-strand units and known as bacterial amyloids (51). It might be that FXII recognizes bacterial exogenous amyloid structures as a pathogen-associated molecular pattern (52). This idea is supported by studies showing that FXII binds and activates on endogenous amyloids and misfolded proteins (53, 54), proposing that FXII-dependent activation of PK is a conserved protective response that recognize and clear non-physiological or damaged host proteins in the extracellular space (55). We have recently shown that different pili of *Streptococcus gallolyticus*—a Gram-positive strain and endocarditis isolate—are involved in binding and activation of contact factors. The adhesin from the pilus binds FXII with high affinity, and we proposed that *S. gallolyticus* may trigger inflammation on the endocardium by activation of host blood coagulation and contact system activation (56). For both, the pilin adhesin Gallo2179 and the major pilin (Gallo2178) several amylogenic regions can be predicted by using “Waltz” an amyloid-prediction tool (57, 58). Nevertheless, whether these proteins are amyloids remains to be investigated.

The Gram-positive group A *Streptococcus* (GAS) bind HK, FXII, and FXI *via* their surface M protein (59), which forms fibrous hair-like structures at the bacterial surface (60), but is not part of a pilus (61). Protein FOG, a fibrinogen-binding M-like protein, and protein G, from Group G streptococci also bind HK, FXII, and FXI (62). Interestingly in this context, immunoglobulin binding domain of the streptococcal protein G also forms amyloid fibrils (63).

Moreover, several adhesins from *Candida* spp. have been demonstrated to bind all contact factors (64).

Factor XII binds to human proteins with amyloid-like properties by the fibronectin type I domain (54); however, the precise binding site for pathogens on FXII are unknown. In HK some interactions have been mapped to D3, D5, and D6 (41).

Thus, so far identified bacterial and fungal proteins that are bound by contact factors have several properties in common, a fibrous hair-like structure, extension from the bacterial cell surface, and function as adhesins by binding of fibrinogen, fibronectin, collagen, or laminin. It remains to be investigated to what extend bacterial amyloid proteins play a role for activation of FXII, as also *Streptococcus mutants or Mycobacterium tuberculosis* display amyloid fimbriae (65, 66).

## Contact System Activation by the Pathogen

Binding and local activation of contact factors at the pathogens surface triggers inflammatory reactions that support the first line in host defense against the invaders. However, eukaryotic and prokaryotic microorganisms can exploit the system and induce its activation by different mechanisms. This may promote invasive spread *via* bradykinin-induced vascular leakage, since inflowing nutrient-rich plasma to the infected tissue site might serve as a route for the disseminating pathogen. Microbial cysteine proteases such as SpeB from GAS (67), staphopain A and B from *Staphylococcus aureus* (68), gingipains from *Porphyromonas gingivalis* (69), and cruzipain from *Trypanosoma cruzi* [for a review see Ref. (70)] can directly liberate kinins from HK (see Table 1). *Aeromonas sobria*, a pathogen causing gastroenteritis and sepsis, secrets a serine protease that activates PK, and also directly cleaves HK as well as LK, thereby producing vascular leakage activity (71).

**Table 1:** Enzymes produced by pathogens that activate or cleave contact factors.

Species	Enzyme	Target	Reference
**Bacteria**			
*Aeromonas sobria*	Serine protease (ASP)	Plasma kallikrein (PK), HK, LK	(71)
*Bacillus stearothermophilus*	Thermolysin	Factor XII (FXII)/PK	(72)
*Bacillus subtilis*	Subtilisin	FXII/PK	
Group A *Streptococcus (Streptococcus pyogenes)*	Cysteine protease (SpeB)	HK	(67)
	Streptokinase-activated plasmin	FXII/PK, HK	(73)
*Porphyromonas gingivalis*	Lysine-specific gingipain (Kgp)	HK	(74)
	Arginine-specific gingipains (RgpA, RgpB)	PK	(69)
*Pseudomonas aeruginosa*	Alkaline phosphatase	FXII	(72, 75)
	Elastase	FXII	
*Serratia marcescens*	56-, 60-, and 73-kD proteinases	FXII	(72)
*Staphylococcus aureus*	Staphopains A and B (ScpA and SspB)	HK	(68)
	V8 proteinase	HK	(72)
*Streptomyces caespitosus*	Proteinase	HK	(72)
*Vibrio cholerae*	Protease	Not known	(76)
*Vibrio parahaemolyticus*	Serine protease	FXII/PK	(77)
*Vibrio vulnificus*?	Metalloprotease	FXII/PK	(72, 78)

**Fungi**			
*Aspergillus melleus*	Proteinase	FXII	(72)
*Candida albicans*	Carboxyl peptidase	FXII/PK	(79)
*Candida* spp.	Aspartic proteases	HK	(80–82)

**Parasites**			
*Fasciola hepatica*	Cysteine proteases	HK	(83)
*Plasmodium chabaudi* and *Plasmodium falciparum*	Falcipain-2Falcipain-3	HK	(84)
*Trypanosoma cruzi*	Cysteinyl-Proteinase (Cruzipain)	HK	(70, 85)
*Schistosoma mansoni*	Secreted enzyme	FXII/PK, HK	(86)

Plasmodium parasites, which cause malaria in the host, generate bradykinin in a different way. They process HK intracellular, probably by cysteine proteases. Thus, by releasing vasoactive peptides, derived from host HK, plasmodium is able to induce vasodilatation and endothelial cell permeability to facilitate parasite survival (84, 87).

Furthermore, extracellular bacterial or fungal proteinases generate proteolytic activity of FXII or PK, thereby producing bradykinin indirectly [see Table 1, for a review see Ref. (44, 72)]. Indirect bradykinin liberation can also be induced by activation of host proteinases, as it has been recently shown for secreted streptokinase, a GAS plasminogen activator (73). Many invasive pathogens exploit plasmin as a virulence factor to degrade fibrin clots, overcome tissue barriers, and evade peptide-derived host immune defenses (88, 89). Contact activation by streptokinase-activated plasmin could explain systemic contact activation and bradykinin liberation seen during invasive streptococcal infection (90). Moreover, dysregulation of the tightly regulated hemostasis by contact system activation may represent another virulence mechanism for streptokinase. Accordingly, the data reveal that GAS isolates from invasive infections trigger an activation of the contact system more potently than strains isolated from noninvasive infections (73).

Hence, activation of the contact system by the pathogen adds another level of complexity to the interaction between pathogen and host during infections.

## Activation of the Contact System in Response to Viral Infections

Besides pro- and eukaryotes, there are few studies describing contact system activation arising from viral infections. Infection of ferrets with influenza A virus results in an increased generation of bradykinin in nasal secretion, suggesting that kinins may contribute to local symptoms of sneezing, nasal congestion, and rhinorrhea (91).

Dengue fever is a rapidly spreading mosquito-borne viral infection often manifests in severe forms. Dengue hemorrhagic fever and dengue shock syndrome can lead to life-threatening complications, including vascular permeability and hemorrhagic manifestations. Reduced serum levels of kininogen were observed in dengue fever patients, which may be due to proteolysis and generation of bradykinin to trigger inflammatory reactions (92).

Human immunodeficiency virus (HIV) progressively damage the immune system, which can lead to endothelial dysfunction and liver damage leading to coagulopathy and over time acquired immunodeficiency syndrome. It was shown that HIV-positive patient have significant prolonged prothrombin time and aPTT (93). Another study showed significant decreased PK activity, but HK concentrations were not significant different between healthy controls and HIV-positive patients (94). Similarly, in patients with HIV–hepatitis B virus co-infections, a significant decrease of PK concentration was measured (95), indicating consumption due to activation.

Hantaviruses are responsible for hemorrhagic fever with renal and pulmonary syndrome, both of which present with edema and hemorrhage. Recently Taylor et al. demonstrated that hantavirus-infected cells trigger activation of the kallikrein–kinin system, revealing a novel mechanism of hantavirus-induced vascular leakage. Incubation of contact factors FXII, PK, and HK with hantavirus-infected endothelial cells leads to an increased cleavage of HK, increased amounts of activated FXII and PK, and liberation of bradykinin. In addition, cell permeability could be avoided using inhibitors that directly block bradykinin binding, the activity of FXIIa, or the activity of PK. Furthermore, they first demonstrated a FXII binding and autoactivation on hantavirus-infected endothelial cells (96).

It has not been shown yet whether the virus will be bound and activate contact factors directly. But enveloped viruses probably provide an appropriate surface for contact activation as the viral envelop is typically derived from host cell membranes. Herpes simplex virus 1 (HSV-1) contains phosphatidylserine and tissue factor on its surface, both derived from the host cell membrane. After addition of HSV-1 to plasma, clotting was induced by the extrinsic and intrinsic pathway of coagulation (97), similarly to procoagulant microvesicles (25).

Thus, there is evidence that the contact system is involved in vascular leakage and inflammatory reactions seen in viral infections. It remains to be investigated whether contact factors bind and activate on the viral surface or on viral infected cells, and whether this interaction may protect from virus-induced disease.

## The Role of Contact System Factors in Sepsis

Sepsis is the archetypical disease state were systemic contact activation occurs (6, 98), and multiple animal studies were done targeting the system to evaluate potential therapeutic options. In animal studies with different species pharmacological interventions that inhibit FXII, PK, or bradykinin-receptors during sepsis implicate beneficial for the host; however, human trials still lack the same confidence [for a review see Ref. (99)]. Moreover, little studies exist, revealing the role of single contact factors during microbial sepsis, using knockout animals or specific inhibitors. In a first study, FXII deficient mice were protected against hypotension induced by LPS, but coagulopathy, inflammatory responses, and lethality were not affected (100). Contrary, mice deficient in HK were resistant to LPS-induced mortality and had significantly reduced circulating LPS levels. Binding of LPS to HK induced cleavage and bradykinin release, proposing that HK—as a major LPS carrier in circulation—plays an essential role in endotoxemia (101). With regard to bacterial infection, a recent study by Stroo et al. show, that FXII deficiency in mice improved survival and reduced bacterial outgrowth, in an airway infection with the Gram-negative *Klebsiella pneumoniae*, but the protecting mechanism is unclear. In contrast, FXII-deficient mice did not show a protective phenotype by using Gram-positive *Streptococcus pneumoniae* in the same infection model (102). Thus, the consequences of activating the contact system within the infection process have yet to be established.

## Conclusion and Outlook

John Hageman was the first patient identified with FXII deficiency, and he suffered from recurrent infections all his life. Beside this case, there are no reports in the literature linking contact protein deficiencies to increased susceptibility for infections. Because there are redundancies in the immune system, it is more than likely that such deficiencies only accidentally will be diagnosed, a phenomenon also seen in complement deficiencies.

Local activation due to contact factor binding on the pathogens surface may be protective against several infections, but activation by the pathogen may trigger systemic reactions that result in detrimental effects for the host.

These findings may offer a great promise for the development of novel therapeutic approaches, potentially complementing existing antibiotic therapies. However, the different mechanisms that trigger a systemic contact activation need to be understood more in detail.

## Author Contributions

All authors listed have made a substantial, direct, and intellectual contribution to the work and approved it for publication.

## Conflict of Interest Statement

The authors declare that the research was conducted in the absence of any commercial or financial relationships that could be construed as a potential conflict of interest.
